# Is popularity of total elbow arthroplasty in the United States decreasing? An analysis of trends, demographics, and indications

**DOI:** 10.1016/j.xrrt.2024.03.005

**Published:** 2024-04-05

**Authors:** Haley McKissack, Anthony R. Karzon, Zaamin B. Hussain, Jacob A. Worden, Kevin Y. Heo, Hayden L. Cooke, Akinade Ojimakinde, Michael B. Gottschalk, Eric R. Wagner

**Affiliations:** a Department of Orthopaedic Surgery, Emory University School of Medicine, Atlanta, GA, USA; b Department of Orthopaedic Surgery, Medical College of Georgia, Augusta, GA, USA

**Keywords:** Total elbow arthroplasty, Rheumatoid arthritis, Osteoarthritis, Distal humerus fracture, Elbow arthritis, Elbow replacement

## Abstract

**Background:**

Total elbow arthroplasty (TEA) is an effective surgical intervention that can be used in the treatment of elbow pathologies including osteoarthritis (OA), complex distal humerus fractures, and rheumatoid arthritis (RA). However, there is a paucity of literature assessing trends in the utilization of TEA. The purpose of this study was to identify trends in TEA utilization in the United States (U.S.) from 2010 to 2018.

**Methods:**

A query of the IBM Watson Health MarketScan Database was performed to identify patients that underwent TEA from 2010 to 2018 using Current Procedural Terminology and International Classification of Disease coding. Patients were stratified based on surgical indication into the following groups: distal humerus fracture/post-traumatic sequelae, RA, OA, and other. Population estimates from the U.S. Census Bureau were used to estimate the annual incidence and procedural trends of primary TEA for each surgical indication. Further stratification evaluated TEA trends based on sex, age, and geographic region.

**Results:**

A total of 6522 primary TEAs were performed between 2010 and 2018. The total annual volume of TEAs performed from the start to the end of this time period decreased by 33%, from 694 to 466 cases. Overall, the majority (53.9%, n = 3514) of all TEAs from 2010 to 2018 were performed to treat distal humerus fractures/post-traumatic sequelae, while 22.3% (n = 1457) were performed for RA, 10.8% (n = 702) for OA, and 13.0% (n = 849) for other. Volume and incidence of TEA decreased over time from 2010 to 2018 regardless of surgical indication, sex, and age. The greatest decreases in volume and incidence in TEAs during the study period was observed for RA (58% and 60%, respectively). The smallest change in volume and incidence of TEA was observed for OA, with a 9% decrease in volume from 57 to 52 cases and 14% decrease in incidence from 0.19 to 0.16 per 1,000,000 people.

**Conclusions:**

The incidence and volume of primary TEAs in the U.S. decreased from 2010 to 2018, regardless of surgical indication, sex, and age. The most common indication for TEA is distal humerus fractures and traumatic sequelae, and RA is now a much less common indication than it was in the past. Understanding the national trends of TEA utilization allows us to visualize changes in practice over time to highlight preferences in the treatment of various elbow pathologies.

Total elbow arthroplasty (TEA) is an effective treatment option for several elbow pathologies, the most common of which include osteoarthritis (OA), rheumatoid arthritis (RA), and unreconstructible distal humerus fractures.^30^^,^^34^^,^^36^^,^^38^^,^^45^ TEA is advantageous in that it aims to restore function including range of motion while improving pain and avoiding instability.^36^^,^^45^ In addition, early studies suggested implant survivorship rates from 89.5% to 96% between 1994 and 2006, supporting the longevity of TEA.^6^^,^^31^ Between 1993 and 2011, the volume of TEA performed more than doubled in the United States (U.S.).^5^^,^^36^^,^^40^

Despite its benefits, when compared to lower-extremity arthroplasty procedures, TEA is associated with higher rates of complications, including infection, aseptic loosening, periprosthetic fracture, mechanical failure, and ulnar neuropathy.^23^^,^^37^^,^^43^^,^^44^ In addition, although peak elbow joint moments differ depending on the task type and direction, it has been recommended to limit patients’ weight bearing on the operative extremity indefinitely to less than 5 kg, making appropriate patient selection critical.^4^^,^^7^ These drawbacks have led to a debate and a lack of consensus as to when TEA is the best treatment option, thereby making the relative indications for TEA controversial.

Early after its introduction, TEA was primarily utilized to treat end-stage inflammatory arthritis secondary to RA.^2^^,^^19^^,^^21^^,^^23^^,^^36^^,^^45^ However, with the advent of disease-modifying antirheumatic drugs, or DMARDs, advanced inflammatory arthritis requiring surgical intervention is less common.^12^^,^^16^^,^^36^^,^^40^ More recently, acute complex distal humerus fractures may require TEA when open reduction internal fixation (ORIF) is not feasible, and many studies have suggested that TEA has lower complication rates and comparable, if not improved, functional outcomes.^12^^,^^40^ For OA, arthroscopic débridement with joint preservation may be an alternative to delay and possibly avoid TEA and also avoid the associated surgical risks and postoperative limitations.^1^^,^^22^^,^^26^^,^^41^

There is a paucity of recent literature assessing trends in TEA prevalence, particularly by surgical indication. The purpose of this study was to analyze trends in the prevalence of primary TEA, both overall and stratified by surgical indication. Secondarily, we aimed to assess trends in TEA stratified by sex, age, and geographic region. We hypothesized that the prevalence of TEA has decreased overall over the past decade, with the greatest relative decrease among patients with RA. By identifying temporal trends in TEA utilization, we can better understand the changes in surgical management of some of the most common elbow pathologies treated with TEA while highlighting surgeon preferences based on patient sex, age, and regional factors.

## Methods

This study was deemed exempt from review by our institutional review board because it utilized publicly available, deidentified data from the commercial claims and Medicare IBM MarketScan Commercial Claims and Encounters and Medicare Supplemental and Coordination of Benefits research databases (Ann Arbor, MI, USA). IBM Marketscan (IBM Corp., Armonk, NY, USA) is a combination of over 250 million deidentified records from private and publicly insured patients, which provides detailed, longitudinal information about their care. This database includes information on inpatient admissions and outpatient care and provides demographic information like age, sex, and region of treatment. While there are advantages to utilizing large administrative databases that allow for evaluation of trends in patient care across the U.S., we acknowledge the inherent disadvantages like reliance on accurate procedural and diagnostic coding. The data from this national insurance database were used in conjunction with population estimates from the U.S. Census Bureau to estimate the annual incidence and procedural trends of primary TEAs. All primary TEA procedures were identified in the MarketScan database using the Current Procedural Terminology code 24363. Patients who underwent nonprimary TEAs were excluded (24370, 24371).

International Classification of Diseases, 9^th^ revision, (ICD)-9 and, 10^th^ revision, ICD-10 codes were used to stratify cases into four groups by indication: distal humerus fractures/post-traumatic sequelae, RA, OA, and other from 2010 to 2018. For this study, cases with ICD codes pertaining to distal humerus fractures/post-traumatic sequelae were categorized together; using the corresponding ICD codes, these patients were categorized into one of the four groups. Next, remaining cases which included an ICD code for RA were identified and categorized as a second group. Ungrouped cases with ICD codes for OA were then classified into a third group, and the remainder of cases were grouped as other. Demographic information, including age, sex, and geographic region of procedure, was collected for all patients.

Statistical analysis was performed using SAS version 9.4 (SAS Institute, Cary, NC, USA). IBM MarketScan discharge weights, constructed from the Public Use Microdata Sample of the American Community Survey, were used to determine national estimates of volumes of the procedures using the Complex Samples function of the IBM SPSS Statistics software (IBM Corp., Armonk, NY, USA). Multiple demographic variables are accounted for in the creation and distribution of these weights to individual patient records. The weighted estimates are intended to account for selection biases that result from only analyzing patients with private insurance coverage and Medicare supplemental plans. It is likely that the patient records in this database do not represent the greater U.S. population. Along with the IBM MarketScan database, population estimates from the U.S. Census Bureau were used to estimate the annual incidence of primary TEA stratified by surgical indication. In addition, volume and incidence were compared by sex, age, and across four statistical geographical regions of the U.S. (Northeast, Midwest, South, and West). The statistical significance of national estimates was assessed using 95% confidence intervals; percent changes were considered statistically significant if no overlap of confidence intervals existed between the years 2010 and 2018.

## Results

A total of 6522 TEAs were performed from 2010 to 2018 (Table I). The volume of TEA performed during this time decreased by 33%, from 694 to 466 cases per year (Table II). Similarly, the incidence of total TEAs performed decreased by 36.9%. Volume and incidence of TEA decreased from 2010 to 2018 regardless of surgical indication, sex, and age.

**Table I:** Overall primary total elbow arthroplasty volume by diagnosis from 2010 to 2018.

Indication	N (%)	95% Confidence interval
Distal humerus fracture, fracture sequelae	3514 (53.88%)	(3209 - 3820)
Rheumatoid arthritis	1457 (22.34%)	(1290 - 1623)
Osteoarthritis	702 (10.76%)	(579 - 826)
Other	849 (13.02%)	(680 - 1017)
Total	6522 (100.0%)	(6207 - 6837)

**Table II:** Primary total elbow arthroplasty volumes and incidences by diagnosis per year

Year	Total				Distal humerus Fx + Fx sequelae				Rheumatoid arthritis				Osteoarthritis				Other			
	Volume		Incidence		Volume		Incidence		Volume		Incidence		Volume		Incidence		Volume		Incidence	
2010	694	(571-817)	2.28	(1.88-2.69)	328	(237-419)	1.08	(0.78-1.38)	215	(146-285)	0.71	(0.48-0.94)	57	(32-82)	0.19	(0.11-0.27)	94	(47-141)	0.31	(0.16-0.46)
2011	728	(620-835)	2.37	(2.02-2.72)	333	(256-410)	1.09	(0.83-1.34)	182	(132-232)	0.59	(0.43-0.76)	119	(69-168)	0.39	(0.23-0.55)	95	(55-134)	0.31	(0.18-0.44)
2012	724	(608-839)	2.34	(1.97-2.71)	330	(248-413)	1.07	(0.80-1.33)	190	(132-248)	0.61	(0.43-0.80)	112	(63-162)	0.36	(0.20-0.52)	91	(50-132)	0.29	(0.16-0.43)
2013	589	(469-708)	1.89	(1.51-2.27)	364	(259-468)	1.17	(0.83-1.50)	109	(67-151)	0.35	(0.22-0.48)	77	(43-112)	0.25	(0.14-0.36)	39	(9-69)	0.13	(0.03-0.22)
2014	456	(355-557)	1.45	(1.13-1.77)	283	(197-370)	0.90	(0.63-1.18)	102	(58-146)	0.32	(0.19-0.46)	57	(28-85)	0.18	(0.09-0.27)	14	(0-28)	0.04	(0.00-0.09)
2015	374	(247-502)	1.18	(0.78-1.59)	222	(127-317)	0.70	(0.40-1.00)	81	(32-131)	0.26	(0.10-0.41)	6	(0-17)	0.02	(0.00-005)	65	(0-135)	0.21	(0.00-0.43)
2016	573	(442-703)	1.80	(1.39-2.21)	383	(268-498)	1.20	(0.84-1.56)	84	(42-126)	0.26	(0.13-0.39)	52	(20-85)	0.16	(0.06-0.27)	54	(17-91)	0.17	(0.05-0.29)
2017	492	(339-645)	1.53	(1.05-2.01)	343	(206-480)	1.07	(0.64-1.50)	97	(37-158)	0.30	(0.11-0.49)	34	(6-62)	0.11	(0.02-0.19)	18	(0-38)	0.06	(0.00-0.12)
2018	466	(325-607)	1.44	(1.01-1.88)	267	(155-380)	0.83	(0.48-1.18)	91	(28-153)	0.28	(0.09-0.48)	52	(15-90)	0.16	(0.05-0.28)	55	(8-103)	0.17	(0.02-0.32)
% change	**−32.9%**		**−36.8%**		**−18.6%**		**−23.4%**		**−57.7%**		**−60.2%**		**−8.8%**		**−14.1%**		**−41.5%**		**−44.9%**	

Incidence is reported per 1,000,000; % change represents the difference from 2010 to 2018. Variables represented as estimates with 95% confidence intervals.

### Surgical indications

**Figure 1:**
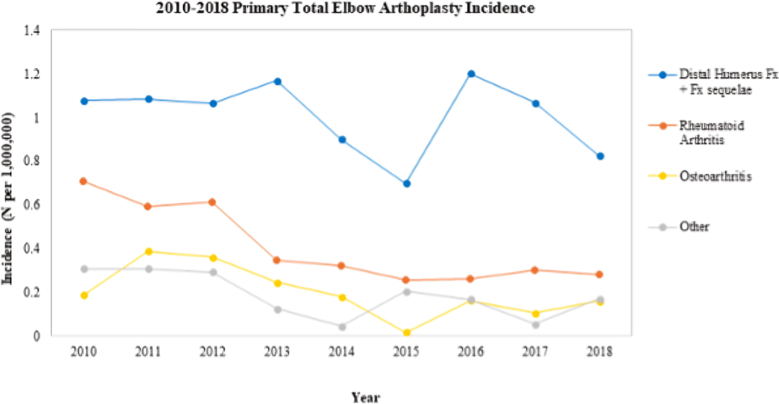
Incidence of total elbow arthroplasty by diagnosis each year between 2010 and 2018.

Table II shows trends in TEA stratified by year and surgical indication. Overall, the majority (53.9%, n = 3514) of TEA from 2010 to 2018 were performed to treat distal humerus fractures/post-traumatic sequelae, while 22.3% (n = 1457) were performed for RA, 10.8% (n = 702) for OA, and 13.0% (n = 849) for other. Volume and incidence of TEA decreased over time, regardless of surgical indication. TEA for RA demonstrated the greatest decrease in volume and incidence, by 58% and 60%, respectively. The smallest change in volume and incidence of TEA was observed for OA, with a 9% decrease in volume from 57 to 52 cases and 14% decrease in incidence from 0.19 to 0.16 per 1,000,000 people. The volume of TEAs performed for distal humerus fractures/post-traumatic sequelae and other decreased, by 19% and 41%, respectively. TEA trends throughout the study period are shown in Figure 1.

### Sex

Comparisons of TEA trends by sex are shown in Table III. Overall primary TEA volumes and incidences decreased from 2010 to 2018 among both males and females. When stratified by surgical indication, decreases in volume and incidence of TEA were observed among males for all indications except distal humerus fracture/post-traumatic sequelae; a 7.7% increase in volume and 1.2% increase in incidence were found between 2010 and 2018 in these patients. Also of note, zero TEAs were performed for males for RA in 2018.

**Table III:** Primary total elbow arthroplasty volumes and incidences by diagnosis and sex

Factor	Volume					Incidence				
	2010		2018		% change	2010		2018		% change
Distal humerus fracture, fracture sequelae										
Male	65	(23-107)	70	(13-127)	7.69	0.44	(0.15-0.72)	0.44	(0.08-0.80)	1.20
Female	263	(182-344)	197	(101-294)	−25.10	1.70	(1.17-2.23)	1.20	(0.61-1.80)	−29.37
Rheumatoid arthritis										
Male	50	(0-100)	0	-	−100.00	0.33	(0.00-0.67)	0.00	-	−100.00
Female	165	(117-213)	91	(28-153)	−44.85	1.07	(0.75-1.38)	0.59	(0.17-0.94)	−44.85
Osteoarthritis										
Male	25	(8-41)	11	(0-31)	−56.00	0.17	(0.06-0.27)	0.07	(0.00-0.20)	−58.65
Female	33	(13-52)	42	(10-73)	27.27	0.21	(0.09-0.34)	0.26	(0.06-0.45)	20.01
Other										
Male	52	(12-93)	5	(0-14)	−90.38	0.35	(0.08-0.62)	0.03	(0.00-0.09)	−90.96
Female	42	(18-66)	51	(4-97)	21.43	0.27	(0.11-0.43)	0.31	(0.03-0.59)	14.50
Total										
Male	192	(114-270)	85	(24-147)	−55.73	1.29	(0.76-1.81)	0.53	(0.15-0.92)	−58.40
Female	502	(404-600)	381	(254-508)	−24.10	3.25	(2.62-3.88)	2.32	(1.55-3.10)	−28.43

Incidence reported per 1,000,000. Variables represented as estimates with 95% confidence intervals.

In contrast to trends among males, the volume of TEA for OA and other increased from 2010 to 2018 among females by more than 20%. TEAs for OA among females increased most, with a 27.3% increase in volume and 20% increase in incidence. TEAs performed for distal humerus fractures/post-traumatic sequelae, as well as for RA, decreased among females, however. Volume and incidence of TEA for distal humerus fractures/post-traumatic sequelae decreased by more than 25%, while volume and incidence for RA decreased by nearly half.

### Age

Comparisons of TEA trends by age group can be found in Table IV. Patients aged 65 years or older underwent the greatest number of TEA, both in 2010 and 2018. Forty percent of all TEAs was performed in this age group in 2010, which increased 56% of all TEAs being performed in this age group in 2018. The fewest number of TEA were performed in patients younger than 45 years both in 2010 and in 2018, with a 56% decrease in incidence in this age group over this 8-year time period.

**Table IV:** Primary total elbow arthroplasty volumes and incidences by diagnosis and age group.

Factor	Volume			Incidence		
	2010	2018	% change	2010	2018	% change
Distal humerus fracture, fracture sequelae						
<45 y	35 (15-55)	12 (0-29)	−65.7	0.19 (0.08-0.30)	0.06 (0.00-0.15)	−66.3
45-54 y	34 (14-55)	26 (0-52)	−23.5	0.77 (0.31-1.24)	0.61 (0.00-1.23)	−20.5
55-64 y	64 (37-90)	39 (6-72)	−39.1	1.87 (1.07-2.63)	0.94 (0.15-1.73)	−49.4
≥65 y	195 (112-278)	190 (87-293)	−2.6	5.03 (2.88-7.18)	3.86 (1.77-5.77)	−23.3
Rheumatoid arthritis						
<45 y	37 (14-60)	0.00	−100.0	0.20 (0.08-0.32)	0.00	−100.0
45-54 y	57 (30-84)	17 (0-36)	−70.2	1.29 (0.68-1.90)	0.40 (0.00-0.84)	−69.0
55-64 y	66 (37-95)	37 (7-68)	−43.9	1.93 (1.08-2.77)	0.90 (0.16-1.62)	−53.5
≥65 y	55 (2-108)	37 (0-88)	−32.7	1.42 (0.05-2.79)	0.75 (0.00-1.74)	−47.1
Osteoarthritis						
<45 y	2 (0-5)	4 (0-12)	100.0	0.01 (0.00-0.03)	0.02 (0.00-0.06)	0.0
45-54 y	16 (3-30)	24 (0-52)	50.0	0.36 (0.07-0.67)	0.56 (0.00-1.23)	56.0
55-64 y	25 (9-41)	10 (0-25)	−60.0	0.73 (0.27-1.18)	0.24 (0.00-0.60)	−66.8
≥65 y	14 (0-28)	14 (0-34)	0.0	0.36 (0.00-0.73)	0.28 (0.00-0.66)	−21.3
Other						
<45 y	14 (0-29)	23 (0-47)	64.3	0.10 (0.00-0.16)	0.12 (0.00-0.25)	21.2
45-54 y	9 (0-18)	0.00 -	−100.0	0.20 (0.00-0.41)	0.00 -	−100.0
55-64 y	45 (21-69)	14 (0-36)	−68.9	1.30 (0.60-2.02)	0.34 (0.00-0.86)	−73.9
≥65 y	26 (0-62)	18 (0-52)	−30.8	0.70 (0.00-1.61)	0.37 (0.00-1.03)	−47.8
Total						
<45 y	88 (55-122)	39 (9-70)	−55.7	0.47 (0.29-0.65)	0.21 (0.05-0.37)	−56.4
45-54 y	116 (79-154)	67 (24-109)	−42.2	2.62 (1.78-3.47)	1.56 (0.56-2.56)	−40.4
55-64 y	200 (151-248)	101 (49-153)	−49.5∗	5.82 (4.41-7.23)	2.45 (1.20-3.70)	−57.9∗
≥65 y	290 (185-396)	259 (137-380)	−10.7	7.49 (4.77-10.22)	5.26 (2.79-7.23)	−29.8

Incidence reported per 1,000,000. Variables represented as estimates with 95% confidence intervals.

∗ Represents a statistically significant change.

When assessing age groups by surgical indication, varying trends are notable. The lowest percent decrease in TEA from 2010 to 2018 was among patients aged 65 years or older, regardless of surgical indication. Within the RA and distal humerus fracture groups, percent decreases in TEA volume and incidence were greatest among young patients, and least among older patients. No patient younger than 45 years underwent TEA for RA in 2018, which is a 100% decrease from 37 patients in 2010. Decreases in volume by 70%, 44%, and 33% were observed in age groups 45-54, 55-64, and ≥65 years old, respectively, with similar trends in decreasing incidence. Like RA trends, volume and incidence of TEA for distal humerus fracture/post-traumatic sequelae decreased the most among those younger than 45 years (66% and 50%, respectively), but only by 3% in volume and 22% in incidence among patients aged ≥65 years.

Volume and incidence of TEAs performed for OA increased in all age groups except in the 55-64 years age group. Of note, an increase was negligible among patients younger than 45 years, with only 2 TEAs performed in 2010 and 4 in 2018. For other, TEA decreased among all age groups except for patients younger than 45 years; volume increased by more than 60%; and incidence by 21% in this group.

### Geographic region

Table V shows changes in TEA volume and incidences by U.S. region. TEA volume and incidence decreased in the Northeast, Midwest, and South, regardless of surgical indication, but increased in the West. The greatest increase in utilization of TEA in the West was for distal humerus fractures; volume increased by more than 35 times, from only 2 TEAs performed in 2001 to 73 in 2018. The volume of TEA in the West also increased for OA and other but decreased by 40% for RA.

**Table V:** Primary total elbow arthroplasty volumes and incidences by diagnosis and U.S. region.

Factor	Volume					Incidence				
	2010		2018		% change	2010		2018		% change
Distal Factor humerus fracture, fracture sequelae										
Northeast	80	(30-130)	27	(0-54)	−66.25	1.47	(0.56-2.40)	0.49	(0.00-0.98)	−66.97
Midwest	168	(97-240)	92	(32-152)	−45.24	2.56	(1.47-3.65)	1.37	(0.48-2.26)	−46.50
South	73	(45-101)	75	(11-139)	2.74	0.66	(0.41-0.91)	0.62	(0.09-1.15)	−5.97
West	2	(0-7)	73	(8-139)	3550.00	0.03	(0.00-0.11)	0.97	(0.10-1.83)	3254.28
Rheumatoid arthritis										
Northeast	75	(22-129)	31	(0-72)	−58.67	1.38	(0.40-2.37)	0.56	(0.00-1.31)	−59.55
Midwest	53	(22-83)	6	(0-18)	−88.68	0.81	(0.34-1.26)	0.09	(0.00-0.26)	−88.94
South	57	(31-84)	36	(6-66)	−36.84	0.52	(0.28-0.76)	0.30	(0.05-0.55)	−42.19
West	30	(10-50)	18	(0-52)	−40.00	0.43	(0.14-0.72)	0.24	(0.00-0.69)	−44.86
Osteoarthritis										
Northeast	10	(0-21)	0	-	−100.00	0.18	(0.00-0.39)	0.00	-	−100.00
Midwest	18	(3-32)	29	(0-59)	61.11	0.27	(0.05-0.49)	0.43	(0.00-0.87)	57.40
South	27	(11-43)	11	(0-26)	−59.26	0.24	(0.10-0.39)	0.09	(0.00-0.22)	−62.71
West	3	(0-8)	13	(0-30)	333.33	0.04	(0.00-0.11)	0.17	(0.00-0.40)	298.23
Other										
Northeast	17	(1-32)	9	(0-26)	−47.06	0.31	(0.03-059)	0.16	(0.00-0.46)	−48.19
Midwest	36	(0-74)	5	(0-14)	−86.11	0.55	0.00-1.12)	0.07	(0.00-0.20)	−86.43
South	31	(11-51)	9	(0-20)	−70.97	0.28	(0.10-0.46)	0.07	(0.00-0.17)	−73.43
West	10	(0-22)	34	(0-75)	240.00	0.14	(0.00-0.32)	0.45	(0.00-0.99)	212.45
Total										
Northeast	182	(107-257)	67	(15-119)	−63.19	3.31	(1.94-4.69)	1.19	(0.26-2.12)	−64.05
Midwest	275	(188-362)	132	(63-200)	−52.00	4.13	(2.82-5.44)	1.93	(0.93-2.94)	−53.27
South	188	(142-234)	130	(58-203)	−30.85	1.68	(1.27-2.09)	1.06	(0.47-1.65)	−36.90
West	45	(21-70)	137	(50-224)	204.44	0.65	(0.30-0.99)	1.78	(0.65-2.90)	173.85

Incidence reported per 1,000,000. Variables represented as estimates with 95% confidence intervals.

## Discussion

TEA is an effective treatment for several elbow pathologies, including RA, OA, and fractures of the distal humerus.^30^^,^^34^^,^^38^^,^^45^ Although TEA is a well-known surgical technique, its utility has been debated over time as improved options for medical management and earlier, less-invasive surgical options for elbow pathologies have been identified. Among RA patients, DMARDs have been shown to slow inflammatory arthritic changes.^10^ For primary elbow OA, arthroscopic débridement including osteophyte resection, loose-body removal, and capsular release has become the procedure of choice among some surgeons, as it is minimally invasive and has proven effective without posing any long-term activity restrictions.^1^^,^^3^^,^^20^^,^^22^^,^^26^^,^^27^^,^^41^ Still, literature has supported TEA as a viable option—and a preferred option in the elderly—for complex intra-articular distal humerus fractures although functional limitations and implant loosening remain a concern.^8^^,^^13^^,^^15^^,^^28^ Amid these factors, current national trends in utilization of TEA are unknown. The purpose of this study was therefore to assess national trends in the volume and incidence of TEA, with the goal of providing a reference point for surgeons to understand how the common indications for TEA have changed.

From 2010 to 2018, the overall volume and incidence of TEA decreased by more than 30%. In all years, TEA was performed most for distal humerus fractures and traumatic sequelae. Decreases in volume and incidence of TEA from 2010 to 2018 were observed regardless of surgical indication, although the greatest decreases were observed for RA (57.7% and 60.2%, respectively) and other (41.5% and 44.9%, respectively). The least amount of change occurred in TEAs performed for OA; only 5 fewer TEAs were performed in 2018 than in 2010, reflecting an 8.8% decrease in volume and 14% decrease in incidence.

The decline in the annual volume and incidence of TEA appears to be a relatively recent phenomenon and represents a turning point in the popularity of this procedure. Using the University Health Systems Consortium administrative database, Zhou et al reported 3146 TEAs performed over a 5-year period (2007-2011) at a steady increase around 600-700 per year.^46^ Prior to that, Day et al reported the number of TEAs performed in the U.S. increased at a rate of 6.4% in annual procedural volume and 7.6% in annual growth between 1993 and 2007.^5^ More similar to our findings, a Norwegian Arthroplasty Registry study including procedures from 1994 to 2006 reported an overall decrease in number of procedures by 50% in this time period.^9^ Of note, these authors found a slight increase in TEAs for traumatic procedures.

The greatest reduction in TEA utilization in this study occurred in patients diagnosed with RA, which is consistent with findings in prior studies. In an analysis of German patients from 2005 to 2014, Klug et al found a 17% decrease in TEA for inflammatory arthritis.^21^ In a retrospective review of indications for TEA in New York State, Gay et al found a decrease in the proportion of TEAs performed for RA from 48% in 1997 to 19% in 2006.^12^ These decreases are likely attributable to the increase in the use of DMARDs and a resultant overall decrease in need for surgical intervention, as supported by the RA literature. Harty et al found a decreasing rate of procedures among RA patients between 1995 and 2010, with concomitant increases in tumor necrosis factor inhibitor and methotrexate prescriptions.^16^ Stamp et al noted increased rates of TEA in New Zealand during a period of national DMARD restriction.^33^^,^^35^^,^^42^ These studies further suggest that advancement in the management techniques for RA has played a role in decreasing TEA volume and incidence among these patients. Given known increases in DMARD use over time, the decline in TEA for RA found in the current study will likely continue, as seen in a Finnish registry study from 1995 to 2010.^18^ However, unlike other joints where DMARDs control inflammation well, it is possible the elbow is a “break-out joint” where there is continued synovitis during DMARD usage. Indeed, one group found an increase in TEA for DMARD patients from 2010 to 2019^17^; therefore, further work is needed to clarify if DMARD usage really is responsible for changes in trends in elbow arthroplasty in patients with inflammatory arthritis or merely a confounding and unrelated variable.

The present results also demonstrated that TEA was most frequently performed for distal humerus fractures and fracture sequelae. Although a 19% decrease in volume was observed from 2010 to 2018, it remained the most common indication every year, with the final incidence of 0.83 in 2018 more than doubling that of RA, and more than quadrupling that of OA and other. Although still debated, recent literature has supported TEA falling into favor for distal humerus fractures over ORIF. In a prospective randomized controlled trial comparing TEA to ORIF in patients older than 65 years, McKee et al found significantly shorter OR times and better Mayo Elbow Performance scores at 2 years among patients who underwent TEA, with comparable postop ROM and reoperation rates.^28^ Systematic reviews by Ellwein et al and Githens et al also demonstrated comparable functional outcome scores and ROM among patients undergoing ORIF and TEA, with more postoperative complications observed among patients undergoing ORIF.^8^^,^^13^ Furthermore, TEA has also been shown to have good longevity for distal humerus fractures, as Dehghan et al demonstrated only a 4% revision rate at 12 years for patients over 65 years.^6^ However, as our understanding of fixation constructs and predictors of failure has developed, the recent implementation of modern precontoured implants and locking screws has enabled ORIF as a viable treatment option in osteopenic patients. As long-term data emerge, we may in fact see ORIF with modern implants perform better than TEA.^11^^,^^24^^,^^39^

Given these outcomes, as well as the risks of hardware failure, nonunion, malunion, and elbow stiffness after ORIF, it is not surprising that TEA is still most commonly utilized for distal humerus fractures. This is particularly relevant among older patients, who have poorer bone stock and are prone to osteoporotic fractures. This is also reflected in our results, as the majority of TEA for distal humerus fractures were performed in patients aged 65 years or older. Although absolute TEA volume decreased, the proportion of TEA performed for distal humerus fractures in patients aged 65 years or older increased in comparison to other age groups from 60% in 2010 to 71% in 2018. The incidence of osteoporotic distal humerus fractures has been predicted to increase three-fold by 2030,^29^ and while our results indicate that the overall use of TEA will likely continue to decrease, they also indicate a shift toward the majority of TEAs being utilized for distal humerus fractures in patients aged 65 years or older.

Compared to other, the smallest decrease in volume and incidence of TEA overall was observed for OA. The majority of TEAs for OA were performed in females, and incidence decreased among males but increased among females, which is consistent with prior literature.^12^^,^^19^ TEA affects males more than females in a ratio of 4:1, with strenuous manual labor as a predisposing factor.^14^ Given postoperative restrictions and the emergence of arthroscopy for younger or high-demand patients, this likely also accounts for fewer TEAs being performed for OA in the male population.^25^

In addition to females, TEA utilization for OA also increased among patients younger than 55 years, 65 years, and/or older; in contrast, volume decreased by more than 50% among those aged 55-64 years old. Although reasoning behind these specific age discrepancies is difficult to fully elucidate, volumes may be explained by the known average age at presentation being 50 years old.^14^ Furthermore, among younger patients, the overall low volume of TEAs performed is likely also due to age at presentation, as well as minimally-invasive elbow arthroscopy becoming increasingly recognized as an effective joint-preservation technique. Multiple case series, including those by MacLean et al.,^26^ Adams et al,^1^ and Krishnan et al,^22^ have demonstrated improved pain, functional outcomes, and ROM among patients with OA after arthroscopic procedures, including osteophyte resection, capsulectomy, and ulnohumeral arthroplasty. However, our results demonstrate a minor decrease in TEA for OA over time, elucidating questions about the wide spread utilization of these joint-preservation surgeries.

Our analysis demonstrated that geographically, TEA volume and incidence decreased in all U.S. regions, with the exception of the West. In total, volume increased in the West by 200% and incidence by 175%. When stratified by indication, increases were noted for distal humerus fractures and sequelae, OA, and other. We do not have a plausible explanation for this trend at this time. Given that it contrasts trends found throughout the country, future studies may seek to investigate the specific reasons behind this in order to better characterize and guide TEA indications and understand regional differences in the treatment of various elbow pathologies.

There are limitations to our study that are inherent in any study conducted via an insurance claims database. MarketScan is a group of commercially available U.S. databases, and the majority of the data come from large private employers, but records from managed care organizations, hospitals, and CMS (Medicare) are also included.^32^ The results are therefore subject to a selection bias for patients with higher socioeconomic status, and the true incidence and volume of TEA may be different. In addition, the coding in databases provides limited granularity and detail, which may also lead to deviations from true TEA and population estimates. The data collected from the database solely allow us to interpret trends and patient demographics, but they do not provide any information regarding functional outcomes or patient satisfaction. Furthermore, the most recent data available was limited to 2018, so it is possible that our results are not representative of trends over the past five years.

## Conclusions

In this study, we observed an overall decrease in the incidence and volume of primary TEA in the U.S. from 2010 to 2018, regardless of indication, sex, and age. The most common indication for TEA remains distal humerus fractures and traumatic sequelae, while incidence of TEA for RA decreased by more than 60%. Understanding these national trends in TEA utilization may provide a better picture of the leading indications for TEA between 2010 and 2018 and how these indications have changed over time.

## Disclaimers:

Funding: Funding was not obtained for the purposes of this study.

Conflicts of interest: Michael B. Gottschalk receives institutional research support from Stryker and Konica Minolta. He is a board or committee member of the American Society for Surgery of the Hand. He is an editor for the Journal of Hand Surgery and Surgical Techniques In Orthopaedics. He receives no royalties from any of the companies. No research funding from any company was used for or relevant to this study. Eric R. Wagner receives consulting fees from Stryker, Biomet, Acumed, and Osteoremedies and research support from Arthrex and Konica Minolta. He receives no royalties from any of the companies. No research funding from any company was used for or relevant to this study. The other authors, their immediate families, and any research foundation with which they are affiliated have not received any financial payments or other benefits from any commercial entity related to the subject of this article.

## References

[1] Adams J.E., Wolff L.H., Merten S.M., Steinmann S.P. (2008). Osteoarthritis of the elbow: results of arthroscopic osteophyte resection and capsulectomy. J Shoulder Elbow Surg.

[2] Chou T.A., Ma H.H., Wang J.H., Tsai S.W., Chen C.F., Wu P.K. (2020). Total elbow arthroplasty in patients with rheumatoid arthritis. Bone Joint Lett J.

[3] Cohen A.P., Redden J.F., Stanley D. (2000). Treatment of osteoarthritis of the elbow: a comparison of open and arthroscopic debridement. Arthroscopy.

[4] Dam W.V., Meijering D., Stevens M., Boerboom A.L., Eygendaal D. (2022). Postoperative management of total elbow arthroplasty: results of a European survey among orthopedic surgeons. PLoS One.

[5] Day J.S., Lau E., Ong K.L., Williams G.R., Ramsey M.L., Kurtz S.M. (2010). Prevalence and projections of total shoulder and elbow arthroplasty in the United States to 2015. J Shoulder Elbow Surg.

[6] Dehghan N., Furey M., Schemitsch L., Ristevski B., Goetz T., Schemitsch E.H. (2019). Long-term outcomes of total elbow arthroplasty for distal humeral fracture: results from a prior randomized clinical trial. J Shoulder Elbow Surg.

[7] Duijn R.G., Meijering D., Vegter R.J., Albers F., Boerboom A.L., Eygendaal D. (2023). Elbow joint loads during simulated activities of daily living: implications for formulating recommendations after total elbow arthroplasty. J Shoulder Elbow Surg.

[8] Ellwein A., Lill H., Voigt C., Wirtz P., Jensen G., Katthagen J.C. (2015). Arthroplasty compared to internal fixation by locking plate osteosynthesis in comminuted fractures of the distal humerus. Int Orthop.

[9] Fevang B.T., Lie S.A., Havelin L.I., Skredderstuen A., Furnes O. (2009). Results after 562 total elbow replacements: a report from the norwegian arthroplasty register. J Shoulder Elbow Surg.

[10] Finckh A., Choi H.K., Wolfe F. (2006). Progression of radiographic joint damage in different eras: trends towards milder disease in rheumatoid arthritis are attributable to improved treatment. Ann Rheum Dis.

[11] Galal S., Mattar Y., Solyman A.M.E., Ezzat M. (2020). Locking versus non-locking plates in fixation of extra-articular distal humerus fracture: a randomized controlled study. Int Orthop.

[12] Gay D.M., Lyman S., Do H., Hotchkiss R.N., Marx R.G., Daluiski A. (2012). Indications and reoperation rates for total elbow arthroplasty: an analysis of trends in New York State. J Bone Joint Surg Am.

[13] Githens M., Yao J., Sox A.H., Bishop J. (2014). Open reduction and internal fixation versus total elbow arthroplasty for the treatment of geriatric distal humerus fractures: a systematic review and meta-analysis. J Orthop Trauma.

[14] Gramstad G.D., Galatz L.M. (2006). Management of elbow osteoarthritis. J Bone Joint Surg Am.

[15] Harmer L.S., Sanchez-Sotelo J. (2015). Total elbow arthroplasty for distal humerus fractures. Hand Clin.

[16] Harty L., O'Toole G., FitzGerald O. (2015). Profound reduction in hospital admissions and musculoskeletal surgical procedures for rheumatoid arthritis with concurrent changes in clinical practice (1995-2010). Rheumatology.

[17] Hodo T.W., Wilder J.H., Ross B.J., Cole M.W., Savoie F.H., Sherman W.F. (2022). Trends in total joint arthroplasty among patients with rheumatoid arthritis: the effect of recent disease modifying antirheumatic drug utilization guidelines. J Am Acad Orthop Surg Glob Res Rev.

[18] Jämsen E., Virta L.J., Hakala M., Kauppi M.J., Malmivaara A., Lehto M.U. (2013). The decline in joint replacement surgery in rheumatoid arthritis is associated with a concomitant increase in the intensity of anti-rheumatic therapy: a nationwide register-based study from 1995 through 2010. Acta Orthop.

[19] Jenkins P.J., Watts A.C., Norwood T., Duckworth A.D., Rymaszewski L.A., McEachan J.E. (2013). Total elbow replacement: outcome of 1,146 arthroplasties from the scottish arthroplasty project. Acta Orthop.

[20] de Klerk H.H., Welsink C.L., Spaans A.J., Verweij L.P.E., van den Bekerom M.P.J. (2020). Arthroscopic and open debridement in primary elbow osteoarthritis: a systematic review and meta-analysis. EFORT Open Rev.

[21] Klug A., Gramlich Y., Buckup J., Schweigkofler U., Hoffmann R., Schmidt-Horlohé K. (2018). Trends in total elbow arthroplasty: a nationwide analysis in Germany from 2005 to 2014. Int Orthop.

[22] Krishnan S.G., Harkins D.C., Pennington S.D., Harrison D.K., Burkhead W.Z. (2007). Arthroscopic ulnohumeral arthroplasty for degenerative arthritis of the elbow in patients under fifty years of age. J Shoulder Elbow Surg.

[23] Kwak J.M., Koh K.H., Jeon I.H. (2019). Total elbow arthroplasty: clinical outcomes, complications, and revision surgery. Clin Orthop Surg.

[24] Loisel F., Amar Y., Rochet S., Obert L. (2024). Distal humerus fracture in older patients: ORIF vs. total elbow arthroplasty. Orthop Traumatol Surg Res.

[25] Long H., Liu Q., Yin H., Wang K., Diao N., Zhang Y. (2022). Prevalence trends of site-specific osteoarthritis from 1990 to 2019: findings from the global burden of disease study 2019. Arthritis Rheumatol.

[26] MacLean S.B., Oni T., Crawford L.A., Deshmukh S.C. (2013). Medium-term results of arthroscopic debridement and capsulectomy for the treatment of elbow osteoarthritis. J Shoulder Elbow Surg.

[27] Martinez-Catalan N., Sanchez-Sotelo J. (2021). Primary elbow osteoarthritis: evaluation and management. J Clin Orthop Trauma.

[28] McKee M.D., Veillette C.J., Hall J.A., Schemitsch E.H., Wild L.M., McCormack R. (2009). A multicenter, prospective, randomized, controlled trial of open reduction--internal fixation versus total elbow arthroplasty for displaced intra-articular distal humeral fractures in elderly patients. J Shoulder Elbow Surg.

[29] Palvanen M., Kannus P., Niemi S., Parkkari J. (2010). Secular trends in distal humeral fractures of elderly women: nationwide statistics in Finland between 1970 and 2007. Bone.

[30] Pogliacomi F., Schiavi P., Defilippo M., Corradi M., Vaienti E., Ceccarelli F. (2016). Total elbow arthroplasty following complex fractures of the distal humerus: results in patients over 65 years of age. Acta Biomed.

[31] Prasad N., Ali A., Stanley D. (2016). Total elbow arthroplasty for non-rheumatoid patients with a fracture of the distal humerus: a minimum ten-year follow-up. Bone Joint Lett J.

[32] Pugely A.J., Martin C.T., Harwood J., Ong K.L., Bozic K.J., Callaghan J.J. (2015). Database and registry research in orthopaedic surgery: part I: claims-based data. J Bone Joint Surg Am.

[33] Rai S.K., Aviña-Zubieta J.A., McCormick N., De Vera M.A., Lacaille D., Sayre E.C. (2017). Trends in gout and rheumatoid arthritis hospitalizations in Canada from 2000 to 2011. Arthritis Care Res.

[34] Rajaee S.S., Lin C.A., Moon C.N. (2016). Primary total elbow arthroplasty for distal humeral fractures in elderly patients: a nationwide analysis. J Shoulder Elbow Surg.

[35] Richter M.D., Crowson C.S., Matteson E.L., Makol A. (2018). Orthopedic surgery among patients with rheumatoid arthritis: a population-based study to identify risk factors, sex differences, and time trends. Arthritis Care Res.

[36] Samdanis V., Manoharan G., Jordan R.W., Watts A.C., Jenkins P., Kulkarni R. (2020). Indications and outcome in total elbow arthroplasty: a systematic review. Shoulder Elbow.

[37] Sanchez-Sotelo J. (2011). Total elbow arthroplasty. Open Orthop J.

[38] Schoch B.S., Werthel J.D., Sánchez-Sotelo J., Morrey B.F., Morrey M. (2017). Total elbow arthroplasty for primary osteoarthritis. J Shoulder Elbow Surg.

[39] Shin S.J., Sohn H.S., Do N.H. (2010). A clinical comparison of two different double plating methods for intraarticular distal humerus fractures. J Shoulder Elbow Surg.

[40] Singh J.A., Ramachandran R. (2016). Sex differences in characteristics, utilization, and outcomes of patient undergoing total elbow arthroplasty: a study of the US nationwide inpatient sample. Clin Rheumatol.

[41] Sochacki K.R., Jack R.A., Hirase T., McCulloch P.C., Lintner D.M., Liberman S.R. (2017). Arthroscopic debridement for primary degenerative osteoarthritis of the elbow leads to significant improvement in range of motion and clinical outcomes: a systematic review. Arthroscopy.

[42] Stamp L.K., Haslett J., Chapman P., O'Donnell J., Raja R., Rothwell A. (2017). Rates of joint replacement surgery in New Zealand, 1999-2015: a comparison of rheumatoid arthritis and osteoarthritis. J Rheumatol.

[43] Voloshin I., Schippert D.W., Kakar S., Kaye E.K., Morrey B.F. (2011). Complications of total elbow replacement: a systematic review. J Shoulder Elbow Surg.

[44] Welsink C.L., Lambers K.T.A., van Deurzen D.F.P., Eygendaal D., van den Bekerom M.P.J. (2017). Total elbow arthroplasty: a systematic review. JBJS Rev.

[45] Zhang D., Chen N. (2019). Total elbow arthroplasty. J Hand Surg Am.

[46] Zhou H., Orvets N.D., Merlin G., Shaw J., Dines J.S., Price M.D. (2016). Total elbow arthroplasty in the united states: evaluation of cost, patient demographics, and complication rates. Orthop Rev.

